# The history of radiofrequency energy and Coblation in arthroscopy: a current concepts review of its application in chondroplasty of the knee

**DOI:** 10.1186/s40634-018-0168-y

**Published:** 2019-01-14

**Authors:** Scott R. Anderson, Scott C. Faucett, David C. Flanigan, Ralph A. Gmabardella, Nirav H. Amin

**Affiliations:** 1 Department of Orthopaedic Surgery, Loma Linda University Medical Center, 11406 Loma Linda Drive, Suite 218, Loma Linda, CA 92354 USA; 2 Department of Orthopaedic Surgery, Centers For Advanced Orthopaedics, LLC, The George Washington University, 2112 F Street NW, Suite 305, Washington D.C, 20037 USA; 3 Department of Orthopedics, Division of Sports Medicine, Jameson Crane Sports Medicine Institute, The Ohio State University Wexner Medical Center, Suite 2200, Columbus, OH 43202 USA; 4 Kerlan-Jobe Orthopedic Clinic, 6801 Park Terrace, Los Angeles, CA 90045 USA

## Abstract

Radiofrequency energy has had widespread use for a variety of surgical procedures. Its application in orthopedic surgery initiated with shoulder instability. Over the last couple decades it has been applied as surgical tool for cartilage treatment as well. There have been significant gains in its technology and our understanding of its potential benefits. We address its history and advancements in becoming a surgical tool for cartilage lesions along with a review of recent long-term follow up studies.

## Introduction

Articular cartilage within the knee provides a smooth gliding surface that protects subchondral bone by distributing pressures with a low frictional coefficient (Buckwalter 2002). Considering the articular cartilage heals poorly and may potentially lead to osteoarthritis at a younger age, it is critical to provide options within surgery to prevent lesion propagation or to repair the cartilage. Previous retrospective studies of knee arthroscopies demonstrate common incidence of cartilage lesions (Årøen et al. 2004; Curl et al. 1997; Hjelle et al. 2002; Widuchowski et al. 2007). During knee arthroscopy, partial thickness articular cartilage lesions are commonly encountered, with up to 11 % of all knee arthroscopies may be suitable for repair (Årøen et al. 2004). The incidence of treatable defects may be even higher in the athletic population (Flanigan et al. 2010). Though the natural history of cartilage defects is not fully understood, cartilage lesions can lead to degenerative joint disease and are frequently seen with osteoarthritis (Christoforakis et al. 2005).

Currently there is no generally accepted treatment protocol for articular cartilage injuries. International Cartilage Repair Society (ICRS) grade I lesions are superficial and rarely need surgical treatment (Outerbridge 1961). The rationale is the depth and or softening of the cartilage is minimal and the cartilage surface fissuring is superficial. More extensive full thickness lesions (Grade IV) have been treated successfully with micro fracture and cartilage repair and restorative procedures, such as osteochondral autograft transfer, osteochondral allografts, and autologous chondrocyte implantation (Emmerson et al. 2007; Gracitelli et al. 2015; Mithoefer et al. 2007; Mithoefer et al. 2009). Although, there are known operative alternatives for the grade 4 lesions, there is a lack of agreement in addressing partial thickness grade II and III cartilage lesions (Hunziker 2002; Sellards et al. 2002). If the grade 2 and grade 3 lesions are not addressed during surgery, there is a greater risk for continued fissuring, lesion expansion, and potential loose body formation with continued mechanical irritation and effusions (Vangsness Jr et al. 2004; Voloshin et al. 2007).

The treatment of cartilage lesions typically involves the removal of free edges to stabilize the lesion and potentially stimulate healing depending on the depth of the lesion. This debridement has traditionally been performed with mechanical shaving. Mechanical chondroplasty has been shown to provide patient benefit (Anderson et al. 2017) but it can lead to persistent fissures and uneven surface topography (Edwards et al. 2008). Furthermore, over resection of potentially healthy cartilage (Caplan et al. 1997; Mandelbaum et al. 1998) could further damage the joint causing the lesion to progress with time (Baumgaertner et al. 1990; Bert and Maschka 1989).

There are alternative resection methods that involve radiofrequency energy (RFE) or laser applications as described by Barber et al. (2002). The use of RFE for cartilage application has evolved over time from thermal energy to its current form using plasma energy fields for debridement. The original thermal energy designs lead to articular injury (Lu et al., 2001) which prompted the development of newer technology as seen with plasma. Plasma applications on cartilage can create a more uniform articular surface which provides an improved gliding surface (Wienecke and Lobenhoffer 2003).

## History of RFE

The technology of RFE has been available for years in a variety of specialty procedures. Specifically the thermal energy is used to ablate abnormal tissue. Introduced in the 19th century to create neural tissue lesions (Cosman et al. 1984), it has gained usefulness in the fields of cardiology, neurology, oncology and proctology (Brodkey et al. 1964; Daoud and Morady 1995; Kapp et al. 1994; Lesh 1993; Moraci et al. 1992; Seegenschmiedt and Sauer 1992). Though it was extensively studied in tissues of the cardiac and nervous systems, limited evidence was available for musculoskeletal tissues. One of the first histological studies for application in orthopedics was performed on joint capsular specimens from adult sheep. Lopez et al. used different RF intensities found a direct relationship between temperature and percentage of area affected. Overall there was increase in cross-sectional size of fibril diameters of collagen (Lopez et al. 1998). This was found to effectively shrink soft tissues that were exposed to RF energy. Using animal studies as a platform, thermal energy initially entered orthopedics through lasers.

## First applications in orthopedics

Thermal energy was being used prior in orthopedic surgery but predominantly used through lasers. There were several considerable drawbacks to use of laser in orthopedics including cost, safety to surrounding tissue and instrument size. RF provided a newer, safer and more convenient use then laser technology.

The development RFE technique was first used in orthopedics to decrease laxity of soft tissues around joints, specifically in the shoulder instability (Kosy et al. 2011). The rationale was to achieve temperatures of 70 to 80 °C, which would shrink treated collagen and stimulated a healing response, similar to what Lopez discovered in the sheep models (Hayashi and Markel 2001). This treatment, however, did not prove to have long-term success and many cases of instability treated with capsulorraphy alone had continued instability or required additional intervention, with studies noting a 37% failure rate at a 38 month follow-up (D'alessandro et al. 2004; Hawkins et al. 2007). Anderson et al. assessed the risk factors associated with early failure of thermal capsulorraphy which were prior history of operation and multiple recurrent dislocations (Anderson et al. 2002). Furthermore, histologically it was found that the collagen structures were morphologically abnormal for up to 16 months after surgery (McFarland et al. 2002). Therefore, the enthusiasm for RF technology was tempered based on the poor efficacy of its use on capsule and cartilage within the shoulder.

## Use in cartilage pathology

Although its application for instability did not have the lasting affect, use of plasma layer in chondroplasty was a novel method to treat cartilage lesions. Historically, these lesions were treated non-operatively, potentially leading to propagating fissures and further erosion of the cartilage (Vangsness Jr et al. 2004; Voloshin et al. 2007). The use on cartilage was found to have different physical and chemical properties as the probe tip creates a plasma layer through the conductive medium (Voloshin et al. 2007). The energy is converted to heat through molecular friction as the electrolytes in the solution oscillate (Meyer et al. 2005). As the collagen is heated, the triple helix of cartilage changes form. As it cools, the fibers realign in a fashion parallel to the joint (Piez 1984; Shellock and Shields Jr 2000). Additionally bipolar plasma layer has been found to have the added benefit of annealing which make the cartilage surface less permeable (Uthamanthil et al. 2006). The annealing process seals the passage of joint fluid enzymes into subchondral bone and maintain cartilaginous water content. This new layer may provide an impermeable surface that is more resilient to shear stresses commonly encountered in the knee, preventing fissure propagation (Gambardella et al. 2016), most helpful in treating grade 2 and 3 cartilage lesions.

**Fig. 1:**
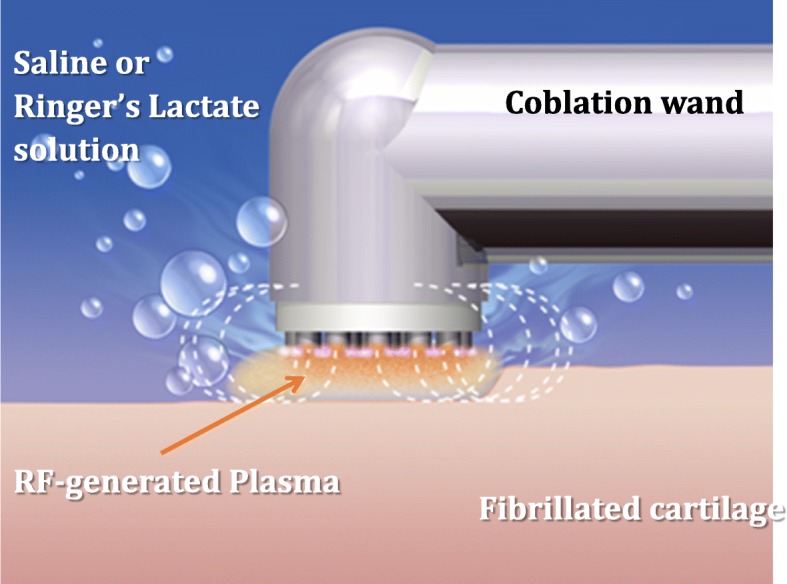
Coblation Plasma layer Mechanism of Action

**Fig. 2:**
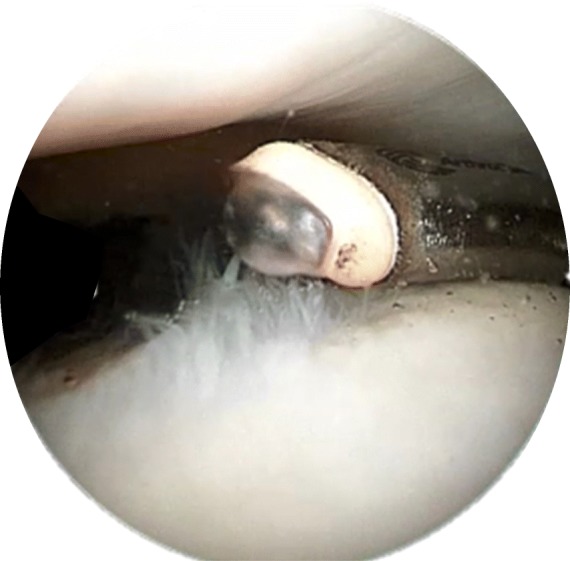
Intaoperative Image of Coblation and depiction of plasma layer

One of the first applications was seen with Kaplan et al., who obtained 6 knee arthroplasty sections and separated the samples into a control and 2 treatment groups (Kaplan et al. 2000). The first treatment group was normal cartilage area and the other area had partial thickness chondromalacia. Both treatment arms were exposed to plasma layer for seconds, incubated, and the chondrocyte viability was evaluated with microscopy. The conclusion was no viable effect on the chondrocytes adjacent to areas of treatment with no visible change in collagen or extracellular matrix compared to untreated areas. Additionally there was smoothing of prior injured areas without extension of fibrillation. Therefore, plasma layer may be adjunct treatment, which is an effective and safe treatment on articular cartilage, especially in cartilage lesions with demonstrated fissures such as grade 2 and grade 3 lesions. Figure 1 demonstrates coblation mechanism of action and Fig. 2 demonstrates intraoperative image of coblation being used in knee.

## Controversy with cartilage viability

Concerns about viability of cartilage cells while using plasma layer prompted several investigations. Lu et al. (2001) evaluated the use of various types of bipolar and monopolar plasma layer devices on bovine femoral osteochondral sections. Within the study, a mechanical jig was used in three treatment groups (contact, non-contact, smoothing). The study evaluated all of the samples with confocal microscope immediately after treatment to evaluate chondrocyte viability. Significant chondrocyte death was found with all devices in all methods of use. However, significant limitations were present in the study design. The energy for certain devices were outside the manufacturer-recommended settings for chondroplasty and at high contact pressure. This produced broad thermal spread that does not correlate to desired tissue effects, leading to detrimental changes on the cartilage and surrounding soft tissue (Lu et al., 2001).

Therefore, in order to better understand the settings and contact pressures the chondrocyte viability using bipolar plasma layer compared to monopolar plasma layer, Amiel et al. (2004) compared metabolic activity of fresh bovine knees to both monopolar and bipolar plasma layer in a non-arthritic knee for chondrocyte viability and metabolic activity. They found the plasma layer can have different responses based on its energy settings and application. Bipolar plasma layer had the smallest amount of chondrocyte death (109.4 ± 22.1 μm) compared to monopolar plasma layer (172.3 ± 34.3 μm). The metabolic activities do not appear to be significantly affected by plasma layer treatment, however this was not found to be statistically significant. This was the first study in animals that used the probe for debridement (light contact) rather than “annealing” (prolonged and higher contact). (Table 1 demonstrates the differences between monopoloar and plasma layer.)

**Table 1:** Monopoloar versus Coblation Settings

Conditions used during Cartilage debridement	MONOPOLAR (and most conventional BIPOLAR devices)	COBLATION (plasma based radiofrequency)
Temperature	> 75 °C (will cause chondrocyte death)	25–35 °C (*)
Electrical Current path	Directly passes through tissue: - Monopolar: to a ground plate - Bipolar: from active to return electrode	Not passing directly through tissue: electrical current generates plasma that in turn transfers energy to contact tissue
Voltage setting	300–9000 V	100–350 V (LO mode for cartilage)
Radiofrequency range	0.25–2.5 MHz	100–500 kHz
Contact pressure	Direct contact with tissue	No contact: 1–2 mm away from tissue
Contact time	Short applications	Brush technique reduces likelihood of extended contact

(*) The Ambient wands also feature a fluid temperature alarm triggered at 45 °C

## Plasma layer for cartilage treatment in human subjects

More recently, several long-term follow up studies have been reported with use of coblation for treatment of cartilage lesions on human subjects (Tables 2) (Gharaibeh et al. 2017; Owens et al. 2002; Spahn et al. 2008; Spahn et al. 2010; Spahn et al. 2016a; Spahn et al. 2016b; Voloshin et al. 2007). These studies confirm the safety of plasma layer as a viable option for application in cartilage treatment and has benefits as compared to mechanical chondroplasty.

**Table 2:** Details of clinical studies in knee lesions

Study, Year	Type of study	Knees treated	Follow up	Lesion type	Key results
Gharaibeh et al. (2017)	Level IV, retrospective case series	RFE: 840	Up to 6 months	Most common site of chondral lesion: - Medial femoral condyle (27%) - Patella (21%) - Trochlea (9%)	Postoperative complications: 2.2% Required reoperation: 2.7% Significant improvement in KOOS and WOMAC scores from preoperative to 129 days postoperative (*p* < 0.0001)
Owens et al. (2002)	Level I, RCT	RFE: 20 MD: 19	Up to 24 months	Isolated patellar chondral lesions (Outerbridge Grade II or III)	Fulkerson-Shea score: superior for radiofrequency over mechanical debridement at 24 months (*p* = 0.0006)
Spahn et al. (2008)	Level I, RCT	RFE: 30 MD: 30	Up to 12 months	Cartilage defect(s) of the medial femoral condyle (Outerbridge Grade III)	Significantly better KOOS (*p* < 0.001) and Tegner scores (*p* < 0.001) for RFE over MD at 12 months Significantly lower VAS pain score (*p* = 0.014) for RFE over MD at 12 months
Spahn et al.^a^ (2010)	Level I, RCT	RFE: 25 MD: 15	Up to 48 months	Cartilage defect(s) of the medial femoral condyle (Outerbridge Grade III)	Significantly higher proportion of revisions for persistent knee problems occurred in the MD group than RFE group (4 vs 14; *p* < 0.01) Significantly better KOOS (*p* < 0.001) and Tegner scores (*p* = 0.005) for RFE over MD at 48 months^b^
Spahn et al.^a^ (2016b)	Level I, RCT	RFE: 13 MD: 9	Up to 120 months	Cartilage defect(s) of the medial femoral condyle (Outerbridge Grade III)	Significantly longer mean time to revision for RFE group over MD group at up to 120 months (94.1 vs 62.5 months; *p* = 0.022)
Voloshin et al. (2007)	Level IV, retrospective case series	RFE: 193	Not reported	Partial-thickness articular cartilage lesions (Outerbridge Grade I-IV)	Second-look follow-up arthroscopy of 25 lesions showed 12% with progressive deterioration, 32% with no change, 32% with partial filling of the defect, and 24% with complete filling with stable repair tissue

*Abbreviations*: *KOOS* knee osteoarthritis outcome score, *MD* mechanical debridement, *RCT* randomized controlled trial, *RFE* radiofrequency energy, *VAS* visual analogue scale, *WOMAC* Western Ontario and McMaster Universities Osteoarthritis Index

^a^Follow-up analyses of the initial 2008 analysis by Spahn et al. (2008)

^b^Patients who underwent revision not included in clinical outcomes analysis

The current literature with plasma layer as a viable option for treating cartilage has been noted to exist in animal studies, four level I studies, and several retrospective studies assessing the clinical outcomes, patient satisfaction, radiographic findings, and safety profile. In assessing the histological findings, the literature shows the effects on the cartilage based on technique, surface area coverage, pressure and plasma layers settings in animal models.

Owens et al. published one of the first prospective clinical studies comparing plasma layer to mechanical shaving (Owens et al. 2002). He recruited a total of 39 female patients with isolated and symptomatic patellofemoral lesions. Patients were randomized to mechanical or plasma layer chondroplasty treatment groups and were blinded to treatment type. The plasma layer chondroplasty group underwent coblation therapy at a non-ablative setting. Pre-operative and postoperative Fulkerson-Shea Patellofemoral Joint Evaluation Scores were evaluated for up to 2 years follow up. The preoperative scores were similar between the two groups. Both groups showed improvement with therapy, however, at the 12 and 24-month follow up the plasma layer group showed statically significant better results as compared to the mechanical group.

Voloshin et al. performed a retrospective study on 193 patients that had undergone coblation chondroplasty for partial thickness chondral defects which were Grade 2 and/or Grade 3 (Voloshin et al. 2007). This was the first study that evaluated the effect of plasma layer by second-look procedure by a single surgeon. Of the initial group of 193 patients, 15 patients had a repeat operation within a 38 month period for a new injury or continued pain. There was a total of 25 lesions (11 PF joint, 14 tibiofemoral joint) treated at the time of the index surgery with coblation for ICRS Grade 2 and/or Grade 3 lesions. Twenty-three out of 25 lesions demonstrated grade 3 changes and all were unstable at the time of the initial operation. Initial arthroscopic images from the first surgery were compared to the images of chondral defects at the time of the second procedure. At follow-up arthroscopy, 12% demonstrated progressive deterioration, 32% showed no change, 32% had partial filling of the defect, and 24% showed complete filling with stable repair tissue. No microfracture was performed. The tibiofemoral joint showed statistically better response to coblation than PF lesions.

A randomized trial of 60 patients with grade 3 cartilage defects of the medial femoral condyle with concomitant meniscal injuries compared bipolar RFE to mechanical shaver for treatment of the cartilage defect (Spahn et al. 2008). They were randomly assigned to either procedure and patients were blinded to treatment. The coblation wand was used in a low energy setting and had a sensor to notify the surgeon of temperature exceeding 50^0^ C. Patients returned for a clinical assessment at 6 weeks and 1 year. The authors found significantly better postoperative physical activity (Tegner activity score), decreased subjective knee symptoms (knee and osteoarthritis outcome score (KOOS)) and decreased pain (VAS score) in the plasma layer group compared to mechanical shaver group at all time points. They concluded that RFE is superior to mechanical shaver.

A 4-year follow up study of this study continued to show positive results for plasma layer chondroplasty (Spahn et al. 2010). Fourteen of the original 30 patients in the mechanical group required a second surgery compared to four in the plasma layer group. The mechanical shaver group had statistically higher rates of revision surgeries for related knee symptoms including arthroplasty, osteotomies and repeat arthroscopy. The plasma layer group continued to show better physical activity and subjective knee symptoms. This study reinforced the findings of the initial article supporting the use of plasma layer as compared to mechanical debridement.

Furthermore, long-term follow up of this randomized trial continued to show differences between the mechanical and plasma layer treatment groups. The results of 10-year follow up demonstrated the coblation group had significantly delayed time to revision surgery of approximately 3 years (Spahn et al. 2016b). However, the self-assessment scores were no longer statistically different at 10 years. This is likely attributed to a combination of natural decrease in activity levels and the progression of osteoarthritis.

Gharaibeh et al., published the largest retrospective study to date evaluating the outcome of coblation therapy for cartilage lesions in 824 patients undergoing knee arthroscopy with safety profile and patient outcomes was the endpoints (Gharaibeh et al. 2017). Though many of these 824 patients had meniscal pathology addressed at the time of chondroplasty, the paper addressed the cartilage defect identified at the time of surgery. Of the total patients, 492 patients were followed with KOOS and WOMAC scores in addition to the quality of the chondral and meniscal lesions using the Chondropenia Severity Score. There was a statically significant improvement in self-assessment scores in all patients when comparing the pre and postoperative scores in all self-reported categories. Additional outcome measures demonstrated no associated surgical complications such as osteonecrosis or chondrolysis, which had been previously documented in historical case reports using RFE. Based on this large retrospective series, using plasma layer technology is safe when using the appropriate contact pressures and plasma settings.

It is critical to note, throughout the literature reviewed, the lesions studies have been Grade 2 and 3 lesions with the knee, if the fibrillations have penetrated passed the subchondral plate and/or demonstrate Grade 4 lesions, the efficacy of plasma layer remains unclear.

## Conclusions

Articular cartilage is essential within the knee to distribute pressure and decrease frictional coefficient during movement. Current treatment strategies of articular cartilage injury aim to remove free edges and stabilize the remaining cartilage. This debridement has traditionally been performed with mechanical shaving which risks removing of potentially healthy cartilage. Lesions treated with plasma layer demonstrated improved patient outcomes and reduced incidence of reoperations compared to mechanical shaving. Plasma layer used with appropriate settings and technique can be a safe surgical tool for the treatment of ICRS Grade 2 and Grade 3 lesions within the knee. With the primary of goal of decelerating the progression of cartilage lesions and improved patient outcomes, plasma layer may be a reasonable option to treat patient with Grade 2 and Grade 3 lesions.
